# Cytogenetic analyses of five amazon lizard species of the subfamilies Teiinae and Tupinambinae and review of karyotyped diversity the family Teiidae

**DOI:** 10.3897/CompCytogen.v9i4.5371

**Published:** 2015-10-07

**Authors:** Natália Dayane Moura Carvalho, Federico José Arias, Francijara Araújo da Silva, Carlos Henrique Schneider, Maria Claudia Gross

**Affiliations:** 1 Laboratório de Citogenômica Animal, Universidade Federal do Amazonas, Instituto de Ciências Biológicas, Estrada do Contorno 3000, Aleixo, CEP 69077-000 - Manaus, AM – Brazil; 2 Laboratório de Herpetologia, Universidade de São Paulo, Instituto de Biociências, Rua do Matão, Travessa 14, 321, Cidade Universitária, CEP 05508-090 – São Paulo, SP – Brazil

**Keywords:** Macroteiidae, Chromosome, Heterochromatin, Differential staining, rDNA-FISH

## Abstract

Lizards of the family Teiidae (infraorder Scincomorpha) were formerly known as Macroteiidae. There are 13 species of such lizards in the Amazon, in the genera *Ameiva* (Meyer, 1795), *Cnemidophorus* (Wagler, 1830), *Crocodilurus* (Spix, 1825), *Dracaena* (Daudin, 1801), *Kentropyx* (Spix, 1825) and *Tupinambis* (Daudin, 1802). Cytogenetic studies of this group are restricted to karyotype macrostructure. Here we give a compilation of cytogenetic data of the family Teiidae, including classic and molecular cytogenetic analysis of *Ameiva ameiva* (Linnaeus, 1758), *Cnemidophorus* sp.1, *Kentropyx calcarata* (Spix, 1825), *Kentropyx pelviceps* (Cope, 1868) and *Tupinambis teguixin* (Linnaeus, 1758) collected in the state of Amazonas, Brazil. *Ameiva ameiva*, *Kentropyx calcarata* and *Kentropyx pelviceps* have 2n=50 chromosomes classified by a gradual series of acrocentric chromosomes. *Cnemidophorus* sp.1 has 2n=48 chromosomes with 2 biarmed chromosomes, 24 uniarmed chromosomes and 22 microchromosomes. *Tupinambis teguixin* has 2n=36 chromosomes, including 12 macrochromosomes and 24 microchromosomes. Constitutive heterochromatin was distributed in the centromeric and terminal regions in most chromosomes. The nucleolus organizer region was simple, varying in its position among the species, as evidenced both by AgNO_3_ impregnation and by hybridization with 18S rDNA probes. The data reveal a karyotype variation with respect to the diploid number, fundamental number and karyotype formula, which reinforces the importance of increasing chromosomal analyses in the Teiidae.

## Background

The family Teiidae is composed of lizards formerly known as macroteiids that are restricted to the New World (Giugliano et al. 2007, Harvey et al. 2012). Harvey et al. (2012) recently divided Teiidae in three subfamilies: (1) Teiinae, including the genera *Ameiva* (Meyer, 1795), *Ameivula* (Spix, 1825), *Aurivela* (Bell, 1843), *Aspidoscelis* (Fitzinger, 1843), *Contomastix* (Dumésil and Bibron, 1839), *Cnemidophorus* (Wagler 1830), *Dicrodon* (Dumésil and Bibron, 1839), *Holcosus* (Cope, 1862), *Kentropyx* (Spix, 1825), *Medopheos* (Bocourt, 1874) and *Teius* (Merrem, 1820); (2) Tupinambinae, including the genera *Crocodilurus* (Spix, 1825), *Dracaena* (Daudin, 1801), *Salvator* (Dumésil & Bibron, 1839) and *Tupinambis* (Daudin, 1802); and (3) Callopistinae, which contains the single genus *Callopistes* (Gravenhorst, 1837) (Harvey et al. 2012). However, the phylogenetic hypothesis of Teiidae based on molecular data (Reeder et al. 2002, Giugliano et al. 2007) differs substantially from the hypothesis proposed by Harvey et al. (2012).

Most chromosome data for teiid lizards refer only to the determination of diploid numbers and karyotype formulae (Fritts 1969, Gorman 1970, Lowe et al. 1970, Robinson 1973, Cole et al. 1979, de Smet et al. 1981, Navarro et al. 1981, Ward and Cole 1986, Cole et al. 1995, Markezich et al. 1997, Rocha et al. 1997, Walker et al. 1997, Manriquen-Moran et al. 2000, Veronese et al. 2003). Some species of this family have, however, been analyzed in detail with respect to their chromosomal structure and organization, as revealed by differential staining techniques, such as the detection of heterochromatin and nucleolar organizer regions (NORs), as well as chromosomal physical mapping of DNA sequences (Bickham et al. 1976, Bull 1978, Peccinini-Seale and Almeida 1986, Porter et al. 1991, Rocha et al. 1997, Veronese et al. 2003, Peccinini-Seale et al. 2004, Santos et al. 2007, Santos et al. 2008).

The family Teiidae can be divided into two chromosomal groups: the *Dracaena* group (currently the subfamily Tupinambinae), which has a karyotype with 34–38 chromosomes and a clear distinction of macrochromosomes (M) from microchromosomes (mi), and the *Ameiva* group (currently the subfamily Teiinae), which has a diploid number ranging from 46–56 chromosomes, with no distinction between macrochromosomes and microchromosomes (Gorman 1970).

We did a cytogenetic study of five species in the family Teiidae (*Ameiva ameiva* (Linnaeus, 1758), *Cnemidophorus* sp.1, *Kentropyx calcarata* (Spix, 1825), *Kentropyx pelviceps* (Cope, 1868) and *Tupinambis teguixin* (Linnaeus, 1758)) using classical as well as molecular cytogenetic markers (conventional staining, heterochromatin patterns, NOR locations and chromosomal physical mapping of 18S rDNA sequences). Karyotype organization in the family is discussed.

## Methods

**Figure 1.**
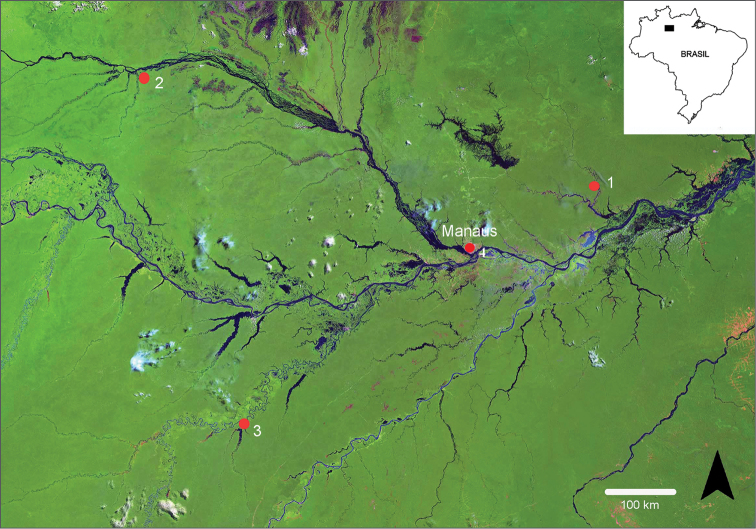
Satellite image of the Amazon basin showing the three different geographical areas; 1 = São Sebastião do Uatumã; 2 = Santa Isabel do Rio Negro; 3 = Tapauá; 4 = Manaus.

Thirty-three specimens belonging to the subfamilies Teiinae and Tupinambinae were collected in the state of Amazonas, Brazil, in the following localities: the riverside forests of the Jatapu river, the city of São Sebastião do Uatumã (0°50' - 01°55'S; 58°50' - 60°10'W), the Darahá and Ayuanã rivers, both in the city of Santa Isabel do Rio Negro (0°24'24"N; 65°1'1"W), the city of Manaus (3°07'13.03"S; 60°01'440"W) and the Purus riverside in the city of Tapauá (5°42'115"S; 63°13'684"W). All of the collections were conducted with permission from the Brazilian Environmental Protection Agency (ICMBio/SISBIO 41825-1). The collection sites are located in public lands (Table 1, Figure 1). The animals were euthanized soon after capture in the field with a lethal dose of the anesthetic sodium thiopental to avoid being deprived of food or water. This research was approved by the Ethics Committee for Animal Experimentation of the Fundação Universidade do Amazonas / Universidade Federal do Amazonas (UFAM) (number 041/2013). No endangered or protected species were used in this research study. The animals underwent cytogenetic procedures and were then fixed with 10% formaldehyde (injected in the coelom and digestive tract), preserved in 70% alcohol. Voucher specimens were deposited in the Herpetological Collection of the Instituto Nacional de Pesquisas da Amazônia (INPA H31712, 33213, 34791, 34841, 35018).

**Table 1.** Species of the Teiinae and Tupinambinae subfamilies: collection sites, number and the analyzed animals and voucher specimens (lots) are listed. AM: Amazonas.

Subfamily	Species	Collection sites	Number and sex the analyzed animals	Voucher specimens (lots)
	*Ameiva ameiva*	São Sebastião do Uatumã, AM Santa Isabel do Rio Negro, AM Tapauá, AM	11 (four males; three females; four without sex identification)	INPA H33213
	*Cnemidophorus* sp.1	Manaus, AM	13 (five males; eight females)	INPA H35018
Teiinae	*Kentropyx calcarata*	São Sebastião do Uatumã, AM	4 (three males; one females)	INPA H31712
	*Kentropyx pelviceps*	Tapauá, AM	3 (three females)	INPA H34841
Tupinambinae	*Tupinambis teguixin*	São Sebastião do Uatumã, AM Tapauá, AM	3 (two females; one without sex identification)	INPA H34791

Cellular suspensions were obtained from the bone marrow was removed soon after the euthanasia of animals in the field using an *in vitro* colchicine treatment (Ford and Hamerton 1956). Constitutive heterochromatin (CH) was detected using barium hydroxide (Sumner 1972) and the NORs were detected using silver nitrate staining (Howell and Black 1980).

Genomic DNA was extracted from muscle tissue using a phenol-chloroform protocol (Sambrook and Russell 2001) and quantified using a NanoDrop 2000 spectrophotometer (Thermo Scientific). 18S rDNA was amplified by polymerase chain reaction (PCR) using primers 18Sf (5’-CCG CTT TGG TGA CTC TTG AT-3’) and 18Sr (5’-CCG AGGACC TCA CTA AAC CA-3’) (Gross et al. 2010). PCR reactions were performed on a final volume of 15 µL, containing genomic DNA (200 ng), 10× buffer with 1.5 mM of MgCL_2_, Taq DNA polymerase (5 U/µL), dNTPs (1 mM), forward and reverse primers (5 mM) and Milli-Q water. The amplification cycles followed these steps: 1 min at 95 °C; 35 cycles of 1 min at 94 °C, 1 min at 56 °C, 1 min 30 s at 72 °C and 5 min at 72 °C.

The PCR product of the 18S rDNA was labeled with digoxigenin-11-dUTP (Dig- Nick Translation mix; Roche), by nick translation according to the manufacturer’s instructions. The antibody anti-digoxigenin rhodamine (Roche) was used for probing the signal. Homologue (DNA probes from the same species) and heterologue (probes of one species hybridized to the chromosome of another) hybridizations were made under stringency conditions of 77% (2.5 ng/µL of 18S rDNA, 50% formamide, 10% dextran sulfate, and 2× SSC at 37 °C for 18 h) (Pinkel et al. 1986). The chromosomes were counterstained with DAPI (2 mg/ml) in VectaShield mounting medium (Vector). The chromosomes were analyzed using an Olympus BX51 epifluorescence microscope and the images were captured with a digital camera (Olympus DP71) using Image-Pro MC 6.3 software. Mitotic metaphases were processed in Adobe Photoshop CS4 software and were measured using program ImageJ software. Chromosomes were organized by decreasing size, and chromosome morphology was determined based on the arm ratio for metacentric (m), submetacentric (sm), subtelocentric (st) and acrocentric (a) chromosomes (Levan et al. 1964). The karyotype formula was determined according to chromosomes that show a gradual series of acrocentric chromosomes, number of biarmed chromosomes, number of uniarmed chromosomes and number of macrochromosomes (M), and microchromosomes (mi) (Lowe and Wright 1966, Peccinini-Seale 1981). Macrochromosomes and microchromosomes are chromosomes that can be differentiated according to size; macrochromosomes are large and have one or two chromosome arms; microchromosomes are small (0.5–1.5 μm), puntiform and do not have any specific chromosome morphology.

## Results

**Figure 2.**
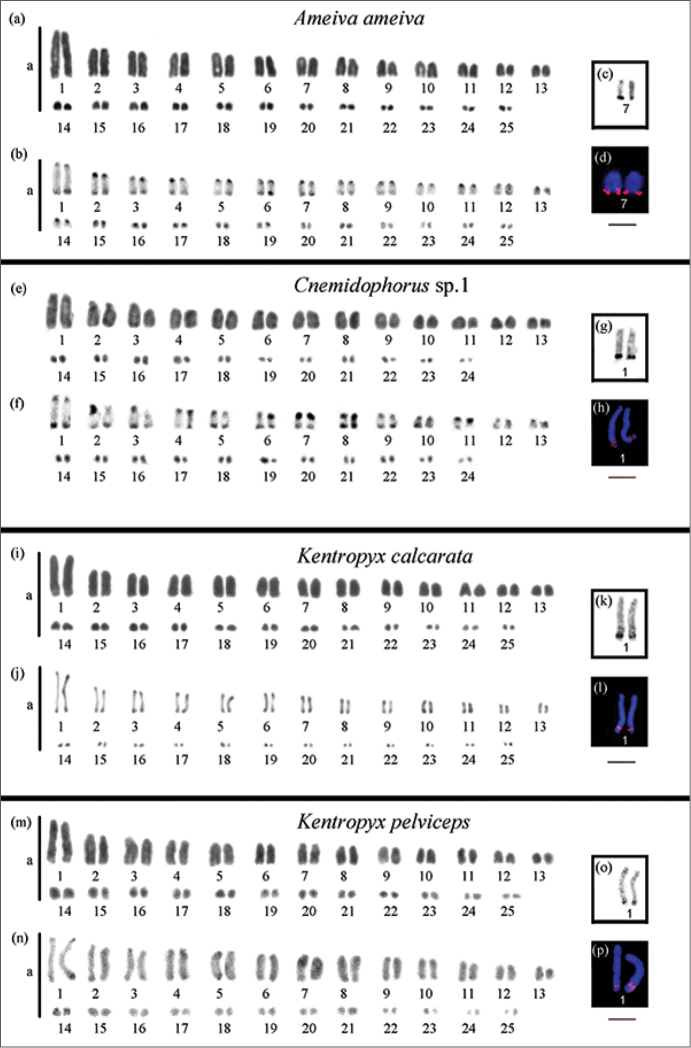
Karyotypes of species belonging to Teiinae: **a, e, i, m** in conventional Giemsa staining **b, f, j, n** Regions of heterochromatin evidenced by C-band technique **c, g, k, o** highlight the nucleolar pair impregnated with AgNO_3_ **d**, **h**, **l**, **p** highlighted in the chromosome pair bearing the site of 18S rDNA (red) and chromosomes were counterstained with DAPI. m = Macrochromossome, mi = microchromossome. Scale bar = 10 µm.

**Figure 3.**
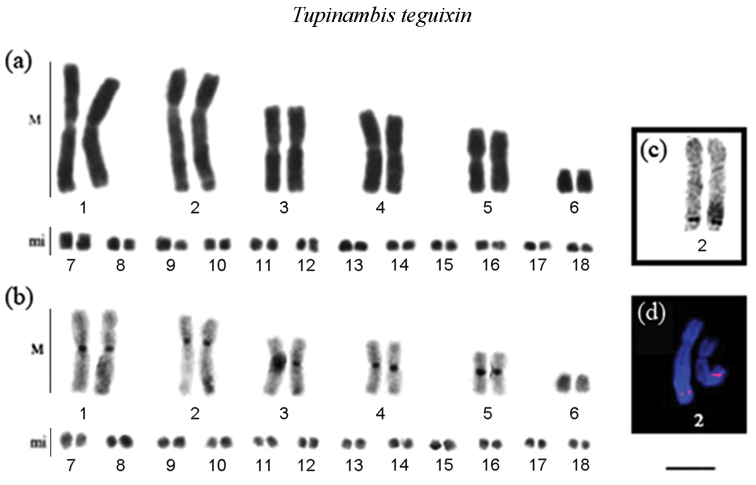
Karyotype of *Tupinambis teguixin*: **a** in conventional Giemsa staining **b** Regions of heterochromatin evidenced by C-band technique **c** highlight the nucleolar pair impregnated with AgNO_3_ **d** highlight the chromosome pair bearing the site of 18S rDNA (red) and chromosomes were counterstained with DAPI. m = Macrochromossome, mi = microchromossome. Scale = 10 µm.

The diploid number for all specimens of *Ameiva ameiva*, *Kentropyx calcarata* and *Kentropyx pelviceps* was 50 chromosomes, and the karyotypic formula was classified by a gradual series of acrocentric chromosomes (Fig. 2a, i and m). *Cnemidophorus* sp.1 had 48 chromosomes with 2 biarmed chromosomes, 24 uniarmed chromosomes and 22 microchromosomes (Fig. 2e). *Tupinambis teguixin* had 36 chromosomes with 12 macrochromosomes (M) and 24 microchromosomes (mi). Pairs 1, 3, 4 and 5 of the macrochromosomes were metacentric and pairs 2 and 6 were submetacentric chromosomes (Fig. 3a). A secondary constriction was observed in the distal region of the long arms of pair 1 in *Cnemidophorus* sp.1, *Kentropyx calcarata* and *Kentropyx pelviceps* and in pair 2 in *Tupinambis teguixin* (Figs 2e, i, m and 3a). No differentiated sex chromosomes were observed in the analysed species.

Constitutive heterochromatin was observed in the centromeric and terminal regions in most chromosomes of *Ameiva ameiva*, *Cnemidophorus* sp.1, *Kentropyx calcarata* and *Kentropyx pelviceps* (Figs 2b, f, j, n). In *Tupinambis teguixin*, heterochromatic blocks were located in the centromeric region of all the macrochromosomes. However, tenuous blocks were observed in the terminal regions in macrochromosomes and microchromosomes (Fig. 3b).

The NORs were located in the terminal region of the long arms of pair 7 in *Ameiva ameiva* (Fig. 2c). In *Cnemidophorus* sp.1, *Kentropyx calcarata* and *Kentropyx pelviceps*, NORs were seen in the distal region of the long arms of pair 1 and in pair 2 in *Tupinambis teguixin*, coincident with the secondary constriction present in the karyotypes of these species (Figs 2g, k, o and 3c, respectively). Fluorescent *in situ* hybridization (FISH) with an 18S rDNA probe revealed a chromosome pair bearing this site, coincident with the NOR sites in all of the five analyzed species (Figs 2d, h, l, p and 3d).

## Discussion

Since the 1970s, cytogenetic analysis of the family Teiidae has shown that individuals could be categorized into two groups: the *Ameiva* group, with diploid number varying from 30–56 chromosomes, with no distinction between macrochromosomes and microchromosomes, and the *Dracaena* group, with a karyotype varying from 34–38 chromosomes, with a clear distinction between macrochromosomes and microchromosomes (Gorman 1970). By the end of the 1980s, several osteological and morphological studies corroborated the chromosomal data, thus supporting these two groups, which were subsequently considered subfamilies (Estes et al. 1988): Teiinae (*Ameiva* group) and Tupinambinae (*Dracaena* group).

Most karyotype data comes from species of the subfamily Teiinae, with descriptions of diploid numbers for 63 species. The karyotypes reveal a diploid number varying from 2n=30 in *Ameiva auberi* (Cocteau, 1838) to 2n=54 in *Teius oculatus* (D’orbigny & Bibron, 1837) and *Teius teyou* (Daudin, 1802), besides the presence of sex chromosomes of XX/XY in *Aspidocelis tigris tigris* (Baird & Girard, 1852) and *Ameivula littoralis* (Rocha, Bamberg Araújo, Vrcibradic, 2000). Some *Aspidoscelis* species show triploid numbers such as *Aspidoscelis tessalatus* (Say, 1823) with 69 chromosomes. Interspecific hybridization has been observed in some species of the genus *Aspidoscelis*, which were previously placed within the genus *Cnemidophorus* (Lowe et al. 1970, Walker et al. 1997, Lutes et al. 2010, Manriquez-Morán et al. 2000). Although the *Ameiva* group proposed by Gorman (1970) corresponds to the subfamily Teiinae, some species have a distinction between macrochromosomes and microchromosomes, while most chromosomes are acrocentric. This finding is contrary to what was proposed by Gorman (1970) as a cytogenetic feature of the *Ameiva* group (Table 2).

**Table 2.** Basic cytogenetic data compiled from the literature for the Teiidae family. Diploid number (2n), karyotypic formula (KF), fundamental number (FN). Three descriptions of karyotypic formulas: (a) number of biarmed chromosomes, number of uniarmed chromosomes and number of microchromosomes; (b) chromosomes that show a gradual series of acrocentric chromosomes; (c) macrochromosome chromosomes (M) and microchromosomes (mi). For data not included in the literature, “-” is indicated.

Subfamily	Genus	Species (*sensu* [2])	Species (initial description)	2n	Type of KF and description	FN	Reference
Callopistinae	*Callopistes*	*Callopistes flavipunctatus*	*Callopistes flavipunctatus*	2n=38	c (12M+26m)	50	2
		*Callopistes maculatus*	*Callopistes maculatus*	2n=38	c (12M+26m)	26, 50	2, 8
Teiinae	*Ameiva*	*Ameiva ameiva*	*Ameiva ameiva*	2n=50	a (0: 26: 24) b (gradual series of acrocentric chromosomes)	50	2, 18
Tupinambinae		*Ameiva auberi*	*Ameiva auberi*	2n=30	a (8: 10:12)	38	11
		*Ameiva chrysolaema*	*Ameiva chrysolaema*	2n=50	a (0: 22: 28), (6: 20: 24)	50, 56	2
		*Ameiva dorsalis*	*Ameiva dorsalis*	2n=50	a (4: 22: 24)	54	2
		*Ameiva exsul*	*Ameiva exsul*	2n=50	a (0: 26: 24)	50	2
		*Ameiva maynardi*	*Ameiva maynardi*	2n=50	a (4: 22: 24)	54	2
	*Ameivula*	*Ameivula nativo* *Ameivula litorralis* *Ameivula ocellifera*	*Cnemidophorus nativo* *Cnemidophorus littoralis* *Cnemidophorus ocellifera*	2n=50 2n=46 (XX/XY) 2n=50	a (5: 19: 24) a (5: 19: 22) b(gradual series of acrocentric chromosomes)	53 51 -	14 9 18
	*Aspidoscelis*	*Aspidoscelis angusticeps*	*Cnemidophorus angusticeps*	2n=44, 46	a (6: 20: 18), a (2: 24: 20)	50, 48	3, 16
		*Aspidoscelis burti*	*Cnemidophorus burti*	2n=46	a (2: 24: 20)	48	3
		*Aspidoscelis calidipes*	*Cnemidophorus calidipes*	2n=46	a (2: 24: 20)	48	3
		*Aspidoscelis ceralbensis*	*Cnemidophorus ceralbensis*	2n=52	-	-	4
		*Aspidoscelis communis*	*Cnemidophorus communis*	2n=46	a (2: 24:20)	48	3
		*Aspidoscelis costatus*	*Cnemidophorus costatus*	2n=46	a (2: 24:20)	48	3
		*Aspidoscelis cozumelae*	*Cnemidophorus cozumelae*	2n=49, 50	a (0: 28: 21), a (11: 19: 20)	49, 61	3, 16
		*Aspidoscelis deppei*	*Cnemidophorus deppei*	2n=50, 52	a (0: 26: 24), a (0: 28: 24)	50, 52	3, 16
		*Aspidoscelis exsanguis*	*Cnemidophorus exsanguis*	3n=69*	-	-	3, 10
		*Aspidoscelis flagellicaudas*	*Cnemidophorus flagellicaudas*	3n=69*	-	-	3
		*Aspidoscelis gularis*	*Cnemidophorus gularis*	2n=46	a (2: 24: 20)	48	3
		*Aspidoscelis guttatus*	*Cnemidophorus guttatus*	2n=52	a (0: 28: 24)	52	3
		*Aspidoscelis hyperythrus*	*Cnemidophorus hyperythrus*	2n=52	a (0: 28: 24)	52	3
		*Aspidoscelis inoratus*	*Cnemidophorus inoratus*	2n=46	a (2: 24: 20)	48	3, 10
		*Aspidoscelis laredoensis*	*Cnemidophorus laredoensis*	2n=46	a (2: 24: 20)	48	4
		*Aspidoscelis lineatissima*	*Cnemidophorus lineatissima*	2n=52	a (0: 28: 24)	52	3
		*Aspidoscelis marmoratus*	*Cnemidophorus marmoratus*	2n=46	a (0: 22: 24)	46	11
		*Aspidoscelis maslini*	*Cnemidophorus maslini*	2n=47	a (14: 13: 20)	49	3
		*Aspidoscelis mexicana*	*Cnemidophorus mexicana*	2n=46	a (2: 24: 20)	48	3
		*Aspidoscelis motaguae*	*Cnemidophorus motaguae*	2n=46	a (2: 24: 20)	48	3
		*Aspidoscelis neomexicanus*	*Cnemidophorus neomexicanus*	2n=46	a (4: 20: 22)	50	3,10
		*Aspidoscelis opatae*	*Cnemidophorus opatae*	3n=69*	-	-	3
		*Aspidoscelis parvisocius*	*Cnemidophorus parvisocius*	2n=46	a (2: 24: 20)	48	3
		*Aspidoscelis rodecki*	*Cnemidophorus rodecki*	2n=50	-	-	1
		*Aspidoscelis sacki*	*Cnemidophorus sacki*	2n=46	a (2: 24: 20)	48	3
		*Aspidoscelis sptemvittatus*	*Cnemidophorus sptemvittatus*	2n=46	a (2: 24: 20)	48	3
		*Aspidoscelis sexlineatus*	*Cnemidophorus sexlineatus*	2n=46	a (2: 24: 20), a (8: 18: 20)	48, 54	3, 5
		*Aspidoscelis sonorae*	*Cnemidophorus sonorae*	2n=46, 3n=69*	a (4: 20: 22)	48	2, 3, 10
		*Aspidoscelis tesselatus*	*Cnemidophorus tesselatus*	2n=46, 3n=69*	a (4: 20: 22)	50	3, 10, 15
		*Aspidoscelis tigris tigris*	*Cnemidophorus tigris tigris*	2n=46(XX/XY)	a (6: 16: 24)	52	2, 10
		*Aspidoscelis tigris aethiops*	*Cnemidophorus tigris aethiops*	2n=46	a (6: 16: 24)	52	3
		*Aspidoscelis tigris estebanensis*	*Cnemidophorus tigris estebanensis*	2n=46	a (6: 16: 24)	52	3
		*Aspidoscelis tigris gracilis*	*Cnemidophorus tigris gracilis*	2n=46	a (6: 16: 24)	52	3
		*Aspidoscelis tigris marmoratus*	*Cnemidophorus tigris marmoratus*	2n=46	a (6: 16: 24)	52	3
		*Aspidoscelis tigris maximus*	*Cnemidophorus tigris maximus*	2n=46	a (6: 16: 24)	52	3
		*Aspidoscelis tigris septentrionalis*	*Cnemidophorus tigris septentrionalis*	2n=46	a (6: 16: 24)	52	3
		*Aspidoscelis ubiparens*	*Cnemidophorus uniparens*	3n=69*	-	-	3, 10
		*Aspidoscelis velox*	*Cnemidophorus velox*	3n=69*	-	-	3
	*Cnemidophorus*	*Cnemidophorus arenivagus*	*Cnemidophorus arenivagus*	2n=50	a (2: 24: 24)	52	9, 13
		*Cnemidophorus arubensis*	*Cnemidophorus arubensis*	2n=50	a (2: 24: 24)	52	9, 13
		*Cnemidophorus cryptus*	*Cnemidophorus cryptus*	2n=50	-	52	9
		*Cnemidophorus gramivagus*	*Cnemidophorus gramivagus*	2n=50	-	52	9
		*Cnemidophorus lemniscatus*	*Cnemidophorus lemniscatus*	2n=50	a (2: 24: 24)	52	2, 3
		*Cnemidophorus murinus*	*Cnemidophorus murinus*	2n=50	a (2: 24:24)	52	4, 3
	*Contomastix*	*Contomastix lacertoides*	*Cnemidophorus lacertoides*	2n=50	a (0: 26: 24)	52	6, 17
	*Kentropyx*	*Kentropyx borckiana*	*Kentropyx borckiana*	2n=50	a (0: 26: 24)	50	12
		*Kentropyx calcarata*	*Kentropyx calcarata*	2n=50	b(gradual series of acrocentric chromosomes)	50	12, 19
		*Kentropyx striata*	*Kentropyx striata*	2n=50	a (0: 26: 24)	50	12
		*Kentropyx paulensis*	*Kentropyx paulensis*	2n=50	b(gradual series of acrocentric chromosomes)	50	18
		*Kentropyx pelviceps*	*Kentropyx pelviceps*	2n=50	b(gradual series of acrocentric chromosomes)	50	20
		*Kentropyx vanzoi*	*Kentropyx vanzoi*	2n=50	b(gradual series of acrocentric chromosomes)	50	18
	*Teius*	*Teius oculatus*	*Teius oculatus*	2n=54	a (8: 28: 18)	62	17
		*Teius teyou*	*Teius teyou*	2n=54	a (8: 22: 24)	62	2
	*Crocodilurus*	-	*Crocodilurus lacertinus*	2n=34	c (12M+22m)	46	2
		*Crocodilurus amazonicus*	*Crocodilurus amazonicus*	2n=34	c (12M+22m)	46	19
	*Dracaena*	*Dracaena guianensis*	*Dracaena guianensis*	2n=38	a (10:2:26)	48	2
	*Tupinambis*	-	*Tupinambis nigropunctatus*	2n=36, 38	a (10: 2: 24), c (16M+22m)	46, 54	2, 7
		*Tupinambis quadrilineatus*	*Tupinambis quadrilineatus*	2n=38	c (12M+26m)	-	19
		*Tupinambis teguixin*	*Tupinambis teguixin*	2n=38, 36	a (10: 0: 28), (12M+24m)	48	7, 19
	*Salvator*	*Salvator merianae*	*Tupinambis merianae*	2n=36, 38	a (10: 0: 26), c (12M+26m)	48, 50	7, 17, 19

* Polyploidy in triploid form (3n). 1 - Fritts 1969; 2 - Gorman 1970; 3 - Lowe et al. 1970; 4 - Robinson 1973; 5 - Bickham et al. 1976; 6 - Cole et al. 1979; 7 - de Smet et al. 1981; 8 - Navarro et al. 1981; 9 - Peccinini-Seale and Almeida 1986; 10 - Ward and Cole 1986; 11 - Porter et al. 1991; 12 - Cole et al. 1995; 13 - Markezich et al. 1997; 14 - Rocha et al. 1997; 15 - Walker et al. 1997; 16 - Manriquen-Moran et al. 2000; 17 - Veronese et al. 2003; 18 - Santos et al. 2007; 19 - Santos et al. 2008; 20 - Present work.

*Ameiva ameiva* and *Kentropyx calcarata*, which belong to Teiinae, have the same diploid number (2n=50 chromosomes). This result corroborates the available data for these species from different localities (Gorman 1970, Beçak et al. 1972, Peccinini-Seale and Almeida 1986, Schmid and Guttenbach 1988, Sites et al. 1990, Veronese et al. 2003, Santos et al. 2007). However, in present study *Ameiva ameiva* and *Kentropyx calcarata* present a gradual series of acrocentric chromosomes characterized by absence of distinction between macrochromosomes and microchromosomes, similar to the results described by Cole et al. (1995) and Santos et al. (2007). The same finding is observed for *Kentropyx pelviceps*, whose cytogenetic characteristics are revealed for the first time in the present study.

Furthermore, karyotypic formulae composed of biarmed chromosomes, uniarmed chromosomes and microchromosomes has been described for *Ameiva ameiva* and *Kentropyx calcarata* and in the other species genera of the subfamily Teiinae (Lowe and Wright 1966, Gorman 1970, Beçak et al. 1972, Peccinini-Seale and Almeida 1986, Schmid and Guttenbach 1988, Sites et al. 1990, Veronese et al. 2003). These data show that some differences may result from different classification parameters adopted by several authors in their chromosomal analyses.

Currently, the genus *Cnemidophorus* is divided into four morphological groups: (1) *Cnemidophorus lemniscatus* including the species *Cnemidophorus arenivagus* (Markezich, Cole & Dessauer, 1997), *Cnemidophorus arubensis* (Lidth de Jeude, 1887), *Cnemidophorus cryptus* (Cole & Dessauer, 1993), *Cnemidophorus flavissimus* (Ugueto, Harvey & Rivas, 2010), *Cnemidophorus gramivagus* (Mccrystal & Dixon, 1987), *Cnemidophorus lemniscatus espeuti* (Boulenger, 1885), *Cnemidophorus lemniscatus gaigei* (Ruthven, 1924), *Cnemidophorus lemniscatus lemniscatus* (Linnaeus, 1758), *Cnemidophorus lemniscatus splendidus* (Markezich, Cole & Dessauer, 1997), *Cnemidophorus pseudolemniscatus* (Cole & Dessauer, 1993), *Cnemidophorus senectus* (Ugueto, Harvey & Rivas, 2010) and *Cnemidophorus* sp. B.; (2) *Cnemidophorus nigricolor* including the species *Cnemidophorus leucopsammus* (Ugueto & Harvey, 2010), *Cnemidophorus nigricolor* (Peters, 1873), *Cnemidophorus rostralis* (Ugueto & Harvey, 2010) and *Cnemidophorus* sp. A; (3) *Cnemidophorus murinus* including the species *Cnemidophorus murinus* (Laurenti, 1768) and *Cnemidophorus ruthveni* (Burt, 1935) and (4) *Cnemidophorus vanzoi* including the species *Cnemidophorus vanzoi* (Baskin & Williams, 1966) (Harvey et al. 2012). It is noteworthy that several new species of this genus have been described, showing that the taxonomy of this genus has not yet been elucidated, which emphasizes the need for morphological and molecular studies in this genus. Cytogenetically, some species of *Cnemidophorus* have 50 chromosomes, composed of biarmed chromosomes, uniarmed chromosomes and microchromosomes (Table 2, Peccinini-Seale and Almeida 1986). However, the karyotype of *Cnemidophorus* sp.1 from Manaus, in Amazonas state, differs from those described for other species of the genus. This species has 2n = 48 chromosomes with the absence of a pair of microchromosomes (Table 2, present study). Non-robertsonian chromosomal rearrangements may be associated with chromosomal evolution of this genus, which favored changes in diploid number (reduction in diploid number). Another population in Amazonas state (county Manacapuru) identified as belonging to *Cnemidophorus lemniscatus* group has the expected diploid number of 50 chromosomes with the presence of biarmed chromosomes and uniarmed microchromosomes (0:26:24) (Sites et al. 1990). Our results show that the specimens we sampled from Manaus are karyotypically distinct from specimens we sampled from Manacapuru so *Cnemidophorus* sp.1 (*Cnemidophorus lemniscatus* group) could represent a new species.

Seven species from the subfamily Tupinambinae, have had their karyotypes analyzed, with diploid numbers varying from 2n=34–38 chromosomes, with the presence of both macrochromosomes and microchromosomes (Santos et al. 2008, present study). No sex chromosome system has been documented in the subfamily (Gorman 1970). *Tupinambis teguixin* has 2n=36 chromosomes (12M+24m) (Table 2) the same number and karyotype formula was found by other authors (Gorman 1970, de Smet et al. 1981, Santos et al. 2008). Beçak et al. (1972) described a diploid number of 38 chromosomes (12M+26m) for *Tupinambis teguixin*, with an additional pair of microchromosomes.

In the family Teiidae, heterochromatic blocks are located in the centromeric and terminal regions of almost all chromosomes. In some chromosomes, heterochromatic blocks are present in the pericentromeric, interstitial and terminal regions (Table 3). In the five species of the family Teiidae analyzed in this study, we observed a significant number of heterochromatic blocks in the centromere and terminal regions in the most of the chromosomes, which is consistent with similar patterns described in the literature.

**Table 3.** Cytogenetic banding data compiled from the literature for the differential Teiidae family. Nucleolar organizer regions (NORs), constitutive heterochromatin (CH), fluorescent *in situ* hybridization (FISH). Locality: Amazonas (AM), Bahia (BA), United States (USA), Espírito Santo (ES), Goiás (GO), Mato Grosso (MT), Minas Gerais (MG), Pará (PA), Rio de Janeiro (RJ), Rio Grande do Sul (RS), Rondônia (RO), São Paulo (SP), Sergipe (SE), Tocantins (TO). For data not included in the literature, “-” is indicated.

Subfamily	Species (Current description)	Species (Initial description)	Locality	NOR	CH	FISH	Reference
Teiinae	*Ameiva ameiva*	*Ameiva ameiva*	GO, RO, MT, TO	Terminal region of the long arms of pair 7	Centromeric and terminal regions	-	8
	*Ameiva ameiva*	*Ameiva ameiva*	AM	Terminal region of the long arms of pair 7	Centromeric and terminal regions	18S rDNA (pair 7)	Present work
	*Ameiva auberi*	*Ameiva auberi*	-	-	-	45S rDNA (pair of microchromosomes)	4
	*Aspidoscelis gularis*	*Cnemidophorus gularis*	USA		Centromeric region	-	1
	*Aspidoscelis laredoensis*	*Cnemidophorus laredoensis*	USA	-	Centromeric region	-	1
	*Aspidoscelis marmoratus*	*Cnemidophorus marmoratus*	-	-	-	45S rDNA (pair 2)	4
	*Aspidoscelis sexlineatus*	*Cnemidophorus sexlineatus*	USA	-	Centromeric region	-	1
	*Aspidoscelis tigris*	*Cnemidophorus tigriss*	USA	-	Centromeric region	-	2
	*Ameivula littoralis* *Ameivula nativo* *Ameivula ocellifera*	*Cnemidophorus littoralis* *Cnemidophorus nativo* *Cnemidophorus ocellifera*	RJ ES BA, SE, MG	Terminal region of the long arms of pair 8 Multiple NORs (not indicated pairs) Terminal region of the long arms of pair 5	- - Centromeric and terminal regions	- - -	7 5 8
	*Cnemidophorus arenivagus*	*Cnemidophorus arenivagus*	-	Terminal region of the long arms of pair 1	-	-	3
	*Cnemidophorus cryptus*	*Cnemidophorus cryptus*	-	Terminal region of the long arms of pair 1	-	-	3
	*Cnemidophorus gramivagus*	*Cnemidophorus gramivagus*	-	Terminal region of the long arms of pair 1	-	-	3
	*Cnemidophorus lemniscatus*	*Cnemidophorus lemniscatus*	-	Terminal region of the long arms of pair 1	-	-	3
	*Cnemidophorus* sp.1	-	AM	Terminal region of the long arms of pair 1	Centromeric and terminal regions	18S rDNA (pair 1)	Present work
	*Contomastix larcetoides*	*Cnemidophorus larcetoides*	RS	-	Centromeric region	-	6
	*Kentropryx calcarata*	*Kentropryx calcarata*	BA, TO, MT	Distal region of the long arms of pair 1	-	-	8
	*Kentropryx calcarata*	*Kentropryx calcarata*	AM	Distal region of the long arms of pair 1	Centromeric and terminal regions	18S rDNA (pair 1)	Present work
	*Kentropryx paulensis*	*Kentropryx paulensis*	SP	Distal region of the long arms of pair 1	Centromeric and terminal regions	-	8
	*Kentropyx pelviceps*	*Kentropyx pelviceps*	AM	Distal region of the long arms of pair 1	Centromeric and terminal regions	18S rDNA (pair 1)	Present work
	*Kentropryx vanzoi*	*Kentropryx vanzoi*	RO	Distal region of the long arms of pair 1	-	-	8
	*Teius oculatus*	*Teius oculatus*	RS	Multiple NORs (not indicated pairs)	-	-	6
Tupinambinae	*Crocodilurus amazonicus*	*Crocodilurus amazonicus*	PA	Distal region of the long arms of pair 2	Pericentromeric region	-	9
	*Salvator meriane*	*Tupinambis merianae*	TO, SP, ES	Distal region of the long arms of pair 2	Pericentromeric region	-	9
	*Tupinambis quadrilineatus*	*Tupinambis quadrilineatus*	GO, TO	Distal region of the long arms of the pair 2	Centromeric, pericentromeric, interstitial, proximal and terminal regions	-	9
	*Tupinambis teguixin*	*Tupinambis teguixin*	GO, TO	Distal region of the long arms of pair 2	-	-	9
	*Tupinambis teguixin*	*Tupinambis teguixin*	AM	Distal region of the long arms of pair 2	Centromeric and terminal regions	18S rDNA (pair 2)	Present work

1 - Bickhan et al. 1976; 2 - Bull 1978; 3 - Peccinini-Seale and Almeida 1986; 4 - Porter et al. 1991; 5 - Rocha et al. 1997; 6 - Veronese et al. 2003; 7 - Peccinini-Seale et al. 2004; 8 - Santos et al. 2007; 9 - Santos et al. 2008

The heterochromatin patterns for *Cnemidophorus* sp.1, *Kentropyx calcarata*, *Kentropyx pelviceps* and *Tupinambis teguixin* are described for the first time in this study. The heterochromatin distributional pattern is similar among the analyzed species, suggesting a common pattern for species in the family Teiidae. Three species in the subfamily Tupinambinae (*Crocodilurus amazonicus* (Spix, 1825), *Salvator merianae* (Duméril & Bibron, 1839) and *Tupinambis quadrilineatus* (Manzani & Abe, 1997), however, show species-specific heterochromatin patterns, with heterochromatic blocks in the centromeric, pericentromeric, interstitial and proximal regions of most chromosomes (Santos et al. 2008). The existence of such a distinctive pattern can likely be attributed to the addition of heterochromatin or the heterochromatization process during the evolution of these species. Heterochromatic regions are rich in repetitive DNA sequences usually located in the centromeric or terminal regions of chromosomes. This has often been considered important species-specific or population markers (Carvalho et al. 2012, Schneider et al. 2013). Even though heterochromatin may be located on the same chromosome region in different species, this does not mean it has the same genetic composition, which may differ in the amount of repetitive DNA sequences in the chromosomes (Chaiprasertsri et al. 2013).

Although the five species in the family Teiidae analyzed in the present study present a conserved karyotype macrostructure, some chromosomal characteristics differentiate the karyotype of these species. In *Cnemidophorus* sp.1, *Kentropyx calcarata*, *Kentropyx pelviceps* and *Tupinambis teguixin*, the presence of a secondary constriction localized in the distal region of pairs 1 and 2 was observed. The secondary constriction is absent in *Ameiva ameiva*.

Secondary constrictions are typically present in a single chromosomal pair and are very common in several lizard species (Bertolloto et al. 1996, Kasahara et al. 1996, Bertolloto et al. 2002, Srikulnath et al. 2009a). This region contain genes that produce ribosomal RNA and these regions may hold nucleoli proteins during the entire process of cellular division (Guerra 1988). In such secondary constrictions, NORs are usually placed and they are identified, indirectly, by silver nitrate impregnation of the chromosomes. Such impregnation marks only nucleoli proteins involved in the transcriptional activity of ribosomal genes of the 45S family. NORs may be located in a single chromosomal pair, a basal characteristic already reported for different lizard species (Porter et al. 1991).

In the present study, the localization of the NORs was revealed as an genus marker and this information has already been discussed for some genera in the family Teiidae, such as *Kentropyx* (*Kentropyx calcarata*, *Kentropyx paulensis* (Boettger, 1893) and *Kentropyx vanzoi* Gallagher & Dixon, 1980), *Crocodilurus* (*Crocodilurus amazonicus*), *Cnemidophorus* (*Cnemidophorus arenivagus*, *Cnemidophorus cryptus*, *Cnemidophorus gramivagus* and *Cnemidophorus lemniscatus lemniscatus*), *Salvator* (*Salvator merianae*) and *Tupinambis* (*Tupinambis quadrilineatus* and *Tupinambis teguixin*). Localization of the NORs is important for characterizing species and evolutionary studies among teiid lizards (Santos et al. 2007, 2008).

*Tupinambis teguixin* has a simple NOR, as evidenced by the secondary constriction of the long arm of pair 2. A common characteristic among species the subfamily Tupinambinae is the presence of such a secondary constriction in pair 2 (Gorman 1970). Four species of the subfamily Teiinae, *Ameiva ameiva*, *Cnemidophorus* sp.1, *Kentropyx calcarata* and *Kentropyx pelviceps*, also have simple NORs, but they are located in distinct chromosomal pairs. In *Cnemidophorus* sp.1, *Kentropyx calcarata* and *Kentropyx pelviceps*, a secondary constriction was seen in pair 1 while in *Ameiva ameiva* occurred in pair 7. The NOR data analyzed for *Ameiva ameiva* and *Kentropyx calcarata* in the present study corroborate previous data (Schmid and Guttenbach 1988, Cole et al. 1995, Veronese et al. 2003, Santos et al. 2007), but for *Cnemidophorus* sp.1 and *Kentropyx pelviceps* they are new data.

Two populations of *Ameiva ameiva* from the eastern Amazon showed multiple NORs involving pairs 1, 2, 6, 16, 18, 19 and some small chromosomes (Peccinini-Seale and Almeida 1986). Some authors suggest that the inter-individual variation observed in *Ameiva ameiva* may be related to the identification of active NOR sites, once the silver nitrate binds to acid nucleoli proteins involved with the transcriptional activity of the ribosomal genes (Miller et al. 1976, Howell and Black 1980, Boisvert et al. 2007). Such variability may also result from impregnation of CH regions rich in acid residues, in which the nitrate impregnates both the NORs and heterochromatic regions not bearing ribosomal sites, thereby not revealing the exact number of NORs (Sumner 2003). Moreover, this variation may be suggesting that *Ameiva ameiva* is a specie complex, as other teiids like *Ameivula ocellifera* (Spix, 1825) (Arias et al. 2011) or *Cnemidophorus lemniscatus* (Harvey et al. 2012).

Using 45S ribosomal DNA probes and FISH, it is possible to understand the organization of the NORs and to elucidate questions concerning the chromosomal organization and karyotypic evolution. The FISH technique is a more refined method than silver nitrate impregnation to locate 45S rDNA sequences in mitotic chromosomes (Carvalho et al. 2012, Terencio et al. 2012, Schneider et al. 2013). However, for the species analyzed in the present study, the fluorescent *in situ* hybridization of the 18S ribosomal gene corroborated the results obtained with silver nitrate impregnation, confirming the existence of this ribosomal site in a single pair of chromosomes. This same pattern was identified in other species in the family Teiidae, supporting the sites seen in a microchromosome pair in *Ameiva auberi* (Cocteau, 1838). In *Aspidoscelis marmorata* (Baird & Girard, 1852), the same pattern was located in a macrochromosome pair (Porter et al. 1991). Furthermore, it was possible to observe a size heteromorphism of the sites between the homologue chromosomes in the four analyzed species, a fact also described for other lizard species (O’Meally et al. 2009, Srikunath et al. 2009b, Srikunath et al. 2011). Such a size heteromorphism is likely associated with unequal crossing-over mechanisms, rearrangements such as transpositions, deletions and/or duplications or variations in the number of rDNA copies present in such regions that would entail some changes in ribosomal sites (Gross et al. 2010, Ribeiro et al. 2008).

## Conclusion

Our present data and those from the literature show that teiid lizards have karyotype variation with respect to diploid number, fundamental number and karyotype formula. This, reinforces the importance to increase the number of chromosomal analyses in the family Teiidae. Studies are currently underway with the chromosomal physical mapping of repetitive DNA sequences in three species of Amazonian teiids that are essential for the understanding of genome organization and karyotype evolution in this group of lizards.
